# Aorta Wall Stress during Exercise in Patients with an Ascending Thoracic Aortic Aneurysm: Insights from a Case Series

**DOI:** 10.1055/a-2558-4266

**Published:** 2025-04-08

**Authors:** Mark J. Haykowsky, Rachel J. Skow, Stephen J. Foulkes, Justin Grenier, John A. Elefteriades, Richard B. Thompson, M. Sean McMurtry

**Affiliations:** 1 Integrative Cardiovascular Exercise Physiology and Rehabilitation (iCARE) Lab, Faculty of Nursing, College of Health Sciences, University of Alberta, Edmonton, Alberta, Canada; 2 Heart, Exercise and Research Trials (HEART) Lab, St Vincent's Institute of Medical Research, Fitzroy VIC, Australia; 3 Department of Radiology and Diagnostic Imaging, University of Alberta, Edmonton, Alberta, Canada; 4 Aortic Institute, Yale University School of Medicine, New Haven, Connecticut; 5 Department of Medicine, Division of Cardiology, Faculty of Medicine and Dentistry, College of Health Sciences, University of Alberta, Edmonton, Alberta, Canada

**Keywords:** thoracic aortic aneurysm, oxygen consumption, aortic wall stress, magnetic resonance imaging

## Abstract

**Background:**

Individuals with ascending thoracic aortic aneurysm (ATAA) are recommended to avoid intense exercise for fear of marked increases in aortic wall stress (AWS). However, no study has measured AWS during exercise. The aim of this case series was to examine AWS during “light-to-moderate” aerobic exercise in individuals with ATAA and healthy control (CON) participants.

**Methods:**

Three clinically stable patients with ATAA (2 male, mean age: 74 ± 1 years) and 3 CON (2 male, mean age: 69 ± 7 years) were studied on 2 separate days. Day 1: a maximal cardiopulmonary exercise test was performed to measure peak aerobic power (VO _2_ peak), maximal heart rate, and blood pressure (BP). Day 2: cardiac and aortic magnetic resonance imaging were performed at rest and during submaximal (3–5 metabolic equivalents) “stepper” exercise during which cardiac output (Qc), aorta diameters, wall thickness, and BP were measured. Circumferential ascending and descending AWS were calculated in accord with LaPlace Law, whereas aorta mechanical efficiency was derived as the AWS/Qc slope.

**Results:**

Patients with ATAA demonstrated lower median VO _2_ peak (18.2 vs. 24.1 mL/kg/min). During exercise, the absolute ascending (ATAA: 257 vs. CON: 269 kPa) and descending AWS increased (ATAA: 224 vs. CON: 207 kPa), and ∆AWS during exercise was similar between ATAA and CON (Ascending, ATAA: 79 vs. CON: 62 kPa; Descending, ATAA: 64 vs. CON: 55 kPa). During exercise, ascending and descending AWS were 76 to 83% below ATAA rupture thresholds (i.e., 800–1,200 kPa) in all patients. Finally, exercise Qc was 17% lower and the ascending AWS/Qc slope was 30% higher in ATAA (16 kPa/L/min) versus CON (12 kPa/L/min).

**Conclusion:**

Our findings demonstrate “light-to-moderate” aerobic exercise produces similar AWS responses between ATAA and CON and is well below aneurysmal rupture thresholds. The higher AWS/Qc slope in ATAA suggests decreased aortic mechanical efficiency and may be a useful measure for exercise prescription for these patients.

## Introduction

Acute aortic dissection is a life-threatening medical emergency that is associated with a high mortality rate. The progressive dilation of the ascending aorta not only increases the risk of these fatal complications but also presents a significant clinical challenge in terms of exercise prescription. 1 While regular physical activity and higher levels of cardiorespiratory fitness, measured objectively as peak aerobic power (VO _2_ peak), are associated with reduced all-cause mortality, 2 there is legitimate concern that acute exercise-induced elevations in blood pressure (BP), especially in patients with ascending thoracic aortic aneurysm (ATAA), may result in excessive increases in aortic wall stress (AWS) that, if repeated chronically, may adversely affect aortic remodeling or result in aortic dissection or rupture. 1

Clinical management of ATAA has relied primarily on aortic diameter measurements to determine disease severity and risk of acute dissection. Intensive pharmacologic BP control has been widely applied in clinical care in order to decrease hemodynamic stress on the aorta wall. 3 However, in accordance with the Laplace Law, AWS is determined by a combination of aortic diameter, wall thickness, and systolic BP (AWS = systolic BP × aorta radius ÷ aorta wall thickness)—yet wall thickness is rarely considered or measured in clinical decision making or risk stratification for ATAA, in part due to the difficulty in measuring this parameter. 4 Indeed, Pape et al 5 demonstrated that nearly 59 and 40% of acute aortic dissections occur in individuals who have aortic diameters below the historical (55 mm) and more contemporaneous (50 mm) elective surgery recommended values. 3 It is possible in some of these individuals that aortic dissection in the presence of a mild-to-moderately dilated aorta may be explained by decreased aortic wall thickness, which, when met with acute increases in systolic BP may result in dissection or rupture—particularly in the presence of abnormal material properties and decreased wall strength. Indeed, a ≥32 kPa/year increase in resting circumferential AWS was associated with an 8.5-fold increase in all-cause mortality over 3 to 5 years, while the change in aortic diameter was not. 6 Consequently, measuring AWS provides a more comprehensive assessment of the mechanical forces that may precipitate acute aortic syndromes. However, to date AWS measurements in individuals with ATAA have been primarily derived using resting aortic diameter, which fails to capture the dynamic mechanical loads experienced by the aortic wall during physical exertion.

Given the importance of regular physical activity, current exercise guidelines for individuals with ATAA recommend “light-to-moderate” intensity aerobic exercise, typically at an absolute exercise intensity equivalent to 3 to 5 metabolic equivalents (METs). 1 3 However, due to concerns that anything above moderate intensity exercise may result in excessive increases in systolic BP (and consequently AWS), most patients with ATAA are counselled against performing intense aerobic exercise, heavy resistance training, and competitive sports, out of concerns that this may increase the risk of acute dissection or rupture and accelerate chronic adverse aortic remodeling. 1 3 This “one-size-fits-all” approach is largely based on expert consensus opinion rather than physiological measurements and fails to account for individual variation in VO _2_ peak and cardiovascular structure and function among patients with ATAA. Consequently, these standardized recommendations may prove too strenuous for deconditioned patients while unnecessarily restricting activity in those with preserved fitness. Moreover, to the best of our knowledge, no study has measured the acute effects of aerobic exercise prescribed within the current guideline recommended levels on AWS, which is essential to provide safe, individualized, and evidence-based exercise prescription in this population.

The aim of this case series study was to examine ascending and descending AWS during guideline-recommended aerobic exercise intensities in individuals with ATAA compared with control subjects with a normal (nonaneurysmal) thoracic aorta (CON). Although descriptive in nature, our “a priori hypothesis” is that acute circumferential AWS during exercise will remain below the ATAA rupture threshold of 800 to 1,200 kPa previously demonstrated by uniaxial strength testing of ATAA tissue. 7 This case series represents a proof of concept for determining thoracic AWS responses to acute aerobic exercise; we provide this critical first step in developing evidence-based, individualized exercise prescriptions for this high-risk population based on acute AWS assessments.

## Materials and Methods

### Study Design

This was a case–control series evaluating VO _2_ peak, hemodynamic, and AWS responses to acute aerobic exercise in individuals with ATAA (cases) versus age- and sex-matched controls (CON) with nonaneurysmal aortas. All participants underwent comprehensive evaluation over 2 days at the University of Alberta Hospital, Edmonton, Canada. Visit 1 consisted of a clinical assessment and a maximal cardiopulmonary exercise test and Visit 2 consisted of a resting and submaximal magnetic resonance imaging (MRI) assessment. All participants were instructed to take their regular medications for both study visits. Details for each procedure are outlined in more detail below. The study was approved by the Local Human Research Ethics Board (Pro 00139002); all participants provided informed consent prior to participating, and all procedures conformed with the Declaration of Helsinki.

### Participant Recruitment and Eligibility

Patients with ATAA were recruited from the aortic disease clinic at the University of Alberta Hospital who met the following inclusion criteria: confirmed diagnosis of unrepaired ATAA (aorta diameter > 40 mm), resting systolic and diastolic BP ≤ 140/≤90 mm Hg, and clinically stable (aneurysm has not grown > 5 mm in the past year, and patients exhibit no indications for surgery as determined by their cardiologist). Control participants were recruited from the local community using advertisements, word of mouth, and contacting participants from previous studies who have provided consent for contact for future studies. Eligible participants were nonhypertensive (BP < 140/90 mm Hg at rest) and had no prior evidence of aortic dilation. Participants in the CON group were recruited to be similar in age and sex compared to the patients with ATAA.

### Study Procedures

#### Clinical Assessment

Height and body weight were assessed using a stadiometer and electronic scale, respectively. Supine resting BP was assessed using an automatic cuff. Three measurements were taken after 10 minutes of quiet rest in a dark room, with the average of all three measurements used for analysis. All participants completed a detailed health and lifestyle questionnaire to obtain demographic, health, and medical history information. Additional information on the patients' medical history and diagnosis was collected from their online medical record.

#### Cardiopulmonary Exercise Test

Participants underwent a maximal cardiopulmonary exercise test with expired gas analysis (TrueOne 2400, Parvo Medics) to quantify VO _2_ peak. The test was performed on an electromagnetically braked upright cycle ergometer (Ergoselect 200, Ergoline GmBH) using a ramp protocol. Following 3 minutes of baseline measurements, participants completed a 3-minute warm-up at 30 Watts, followed by a 15 to 25 Watt/min ramp to volitional exhaustion under Cardiologist supervision. Heart rate and rhythm were monitored continuously from a 12-lead electrocardiogram (ECG; GE Case Stress System, GE HealthCare), and BP was measured at rest and every 2 minutes during exercise using an automatic ECG-gated auscultatory BP monitor (Tango M2 Stress Monitor, Suntech). Participants' subjective rating of perceived exertion (RPE) was also collected every minute using the Borg 6-20 RPE scale. VO _2_ peak was defined as the highest value reached during the test, from a 30-second rolling average of 5-second data epochs. 8

#### Resting and Exercise Magnetic Resonance Imaging

Participants underwent a comprehensive resting and exercise CMR scan (3T Siemens Prisma, Siemens Engineering) to assess cardiac and aortic structure and functional responses to light-to-moderate supine aerobic stepping exercise using an MRI compatible stepping ergometer (Cardio Step Module, Ergospect Medical Technology 9 ). Once all resting structural and functional scans had been performed, participants were then instructed to step in time to an audible metronome at approximately 40 steps/min, with the resistance increased to a level that would achieve 2 to 3 progressive workloads equating to <3 METs and 3 to 5 METs. Imaging commenced after participants had been stepping for approximately 1.5 to 2 minutes to ensure they were in a physiological steady state, with each stage of exercise sustained for approximately 5 to 6 minutes. Heart rate and peripheral capillary oxygen saturation (SpO _2_ ) was monitored continuously, whereas BP was measured every 2 minutes during exercise (Expression MR200, Philips).

#### Aortic Wall Thickness and Diameter

**Fig. 1:**
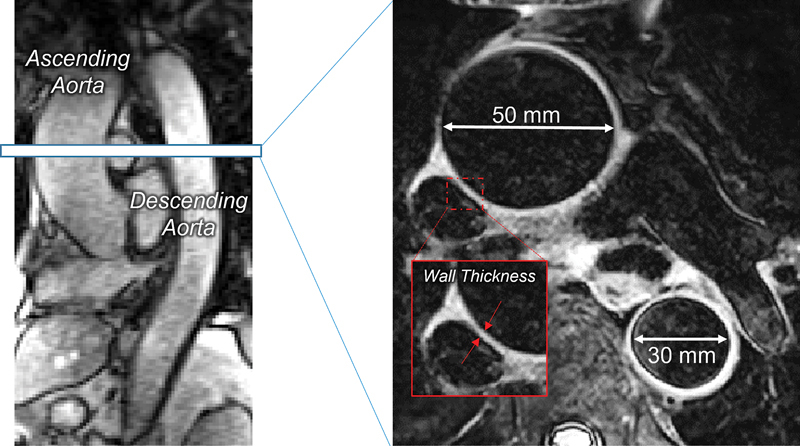
Representative illustration of magnetic resonance imaging assessment of aorta diameters and wall thickness.

Aorta dimensions were obtained in such that the ascending region of the aorta distal to the aortic root and proximal to the aortic arch as well as the thoracic descending aorta were both in view on the same MRI slice ( Fig. 1 ). For patients with ATAA, this represented the region of the aorta where the aneurysm was located, and thus, the largest part of the ascending aorta. Aortic wall thickness was measured with a fat-suppressed black-blood prepared ECG-gated fast spin-echo MRI approach. Imaging parameters: 360 × 220 mm FOV, 432 × 256 acquisition matrix, GRAPPA = 2, TE = 33 ms, 5-mm slice thickness, 0.4 × 0.4 mm reconstructed in-plane spatial resolution ( Fig. 1 ). Imaging of ascending and descending aortic dimensions during exercise were measured using a custom real-time imaging approach with self-gating to reconstruct images at targeted cardiac and respiratory phases, during diastasis and end-expiration. 10 Imaging parameters: radial gradient-echo k-space acquisition, TE = 1.36ms, TR = 2.4 ms, 4-mm slice thickness, 0.86 mm × 0.86 mm reconstructed in-plane spatial resolution, with 60 seconds to total acquisition time ( Fig. 1 ). Aortic diameter was determined as the largest value across the cardiac cycle (i.e., during systole).

#### Rest and Exercise Cardiac Function

Rest and exercise cardiac volumes and hemodynamics *w* ere assessed from a real-time free-breathing steady-state free precession (SSFP) imaging approach (without ECG gating) that has been validated and described in detail previously. 9 In brief, full coverage of both left and right ventricles was provided from a contiguous stack of 23 × 8 mm short-axis slices and 15 × 8 mm horizontal long axis slices, 50-75 frames (36 ms/frame). Imaging parameters are as follows: SSFP pulse sequence with 320 × 260 mm FOV, 128 × 128 acquisition matrix, 50-degree flip angle, GRAPPA = 2, TR = 1.8 ms, TE = 0.9 ms, 2.3 × 2.3 × 8 mm reconstructed voxel size. Biventricular end-diastolic and end-systolic endocardial contours were drawn on short-axis images at end-expiration using in-house software with constant reference to horizontal long-axis images to ensure accurate contouring around the atrioventricular boundaries (RightVol, Leuven, Belgium). End-diastolic and end-systolic volumes were then calculated using the summation of disks method, with stroke volume calculated as the average of left- and right-ventricular stroke volume to account for respiratory variation in stroke volume. Stroke volume was then multiplied by heart rate to derive Qc.

#### Aortic Wall Stress and Mechanical Efficiency Calculations

Circumferential AWS was calculated, as mentioned above, in accordance with LaPlace Law that incorporates systolic BP and aorta geometric parameters. Based on the conservation of mass principle and incompressibility of aortic wall tissue, aortic wall thickness during exercise was back calculated based on resting wall thickness and changes in aortic dimensions during exercise. Each participant's aortic mechanical efficiency was measured as AWS/Qc slope, which was derived from linear regression of their individual resting and exercise AWS and Qc values.

### Statistical Analysis

Given the descriptive nature of this case series, formal statistical hypothesis testing was not performed. Data are presented as median [range] or frequencies.

## Results

### Participant Characteristics

This report consists of three patients with ATAA and three matched CON, and their characteristics are outlined in Table 1 . The patients with ATAA (non-Marfan, nonbicuspid) were receiving antihypertensive treatment and cholesterol-lowering therapy and all participants were normotensive at rest ( Table 1 ).

**Table 1:** Participant characteristics

	CON *N* = 3	ATAA *N* = 3
Male/female	2/1	2/1
Age, y	71 [61–74]	73 [73–75]
Height, cm	178 [160–180]	167 [153–192]
Body mass index, kg/m ^2^	26.6 [23.6–28.2]	27.8 [24.3–31.5]
Resting systolic BP, mm Hg	135 [105–140]	113 [111–124]
VO _2_ peak, mL/kg/min	24.1 [22.6–34.2]	18.2 [17.0–22.5]
Risk factors/comorbidities		
Obesity	0	1
Hypertension	1	3
Dyslipidemia	2	3
Smoking history	2	1
Type 2 diabetes	0	1
Medications		
ACE inhibitor	1	0
Angiotensin receptor blocker	0	3
Beta-blocker	0	1
Calcium channel blocker	0	1
Diuretic	1	0
Statin	2	2
Aorta diameter at rest		
Ascending Aorta (mm)	35.7 [35.6–36.8]	45.5 [41.8–50.4]
Descending Aorta (mm)	28.1 [26.3–29.1]	27.3 [27.0–30.4]

Data are reported as median [range]; BP, blood pressure; VO _2_ peak, peak oxygen consumption; ACE, Angiotensin converting enzyme inhibitor.

### Peak Aerobic Power and Maximal Upright Exercise Responses

All participants completed the maximal cardiopulmonary exercise test (CPET) without any adverse events. Tests were considered a peak effort in all cases—as defined by achieving at least two out of the following criteria: (1) a peak respiratory exchange ratio > 1.10, (2) a peak exercise RPE ≥ 17, and/or (3) ≥85% of their age-predicted maximal heart rate. Individuals with ATAA had lower VO _2_ peak values than CON (18.2 [17.0–22.5] vs. 24.1 mL/kg/min [22.6–34.2]). Furthermore, the VO _2_ peak values for 2/3 patients with ATAA were at or slightly below the threshold for independent living (i.e., ≤18.0 mL/kg/min 11 ) compared with none of the CON participants. This means that for these two individuals with ATAA, activities of daily living (i.e., sweeping, gardening, shoveling snow) may require near maximal effort. Maximal exercise systolic BP values were lower in the ATAA versus CON groups (186 [144–199] vs. 230 mm Hg [202–266]).

### Aortic Wall Stress and its Determinants

**Fig. 2:**
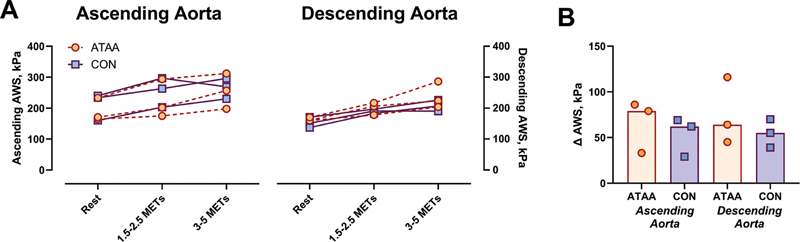
Ascending and descending circumferential aorta wall stress (AWS) at rest and submaximal stepper exercise ( **A** ) in patients with ascending thoracic aortic aneurysm (ATAA) and healthy controls (CON), and the rest to exercise change in AWS ( **B** ) in relation to exercise intensity (determined as metabolic equivalents, METs). Individual data points are shown, while bars on panel **B** represent median values for each group.

Circumferential ascending and descending AWS and the magnitude of exercise-induced changes in AWS were comparable between patients with ATAA and CON during guideline-recommended exercise intensities ( Fig. 2A, B ). This is explained by the fact that while patients with ATAA had a larger ascending aortic diameter (45.5 mm [41.8–50.4 mm] vs. 35.7 mm [35.6–36.8 mm]), they also tended to have a higher ascending aortic wall thickness (1.95 mm [1.54–1.98 mm] vs. 1.33 mm [1.24–1.67 mm]) and lower resting (preexercise) systolic BP (108 [107–120] vs. 125 mm Hg [109–131]). During moderate intensity exercise (i.e., 3–5 METS), both groups showed negligible changes in ascending aortic diameter (ATAA: +0.5 mm [−0.6 to +1.3 mm] vs. CON: +0.6 mm [−0.2 to +0.9 mm]) and wall thickness (ATAA: −0.03 mm [−0.04 to +0.03 mm] vs. CON: −0.03 mm [−0.04 to +0.01 mm]), and comparable increases in systolic BP (ATAA: 29 [25–55] vs. CON: 26 mm Hg [17–42]) resulting in a similar increase in ascending AWS between groups (ATAA: 79 [33–86] vs. CON: 62 kPa [29–69]). Importantly, at 3 to 5 METs, the ascending (ATAA: 257 [198–312] vs. CON: 269 kPa [230–296]) and descending AWS values (ATAA: 224 [204–286] vs. CON: 207 kPa [190–226]) remained well below the established rupture threshold (800–1,200 kPa) in all individuals with ATAA. 7

### Exercise Cardiac Output and Aortic Mechanical Efficiency

**Fig. 3:**
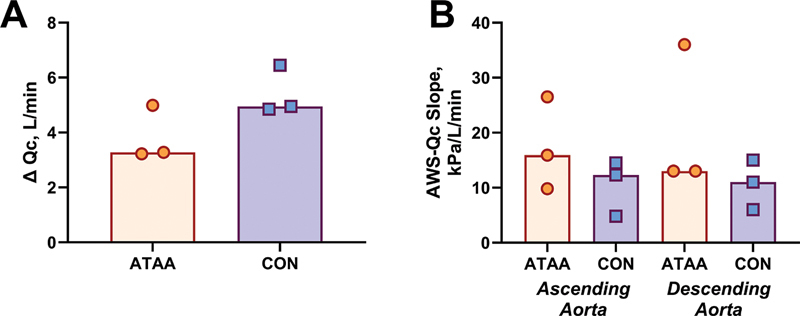
Rest to exercise change in cardiac output (Qc) [ **A** ] and AWS/Qc slope ( **B** ). Individual data points are shown, while bard on panel **B** represent median values for each group. AWS, aorta wall stress.

Qc augmentation during exercise was generally lower in the ATAA versus CON groups ( Fig. 3A, B ). This meant that the AWS/Qc slope tended to be higher in the ATAA group for both the ascending (ATAA: 16 [10–27] vs. CON: 12 kPa/L/min [5–15]) and descending aorta (ATAA: 13 [13–36] vs. CON: 11 kPa/L/min [6–15]), suggesting impaired aortic mechanical efficiency during guideline recommended exercise.

## Discussion

To our knowledge, this is the first report to directly measure circumferential ascending and descending AWS during submaximal exercise in individuals with ATAA. The major novel finding from this proof-of-concept case series is that AWS can be determined in patients with ATAA during aerobic exercise. Importantly, the AWS values we measured during current American Heart Association/American College of Cardiology Joint Committee guideline-recommended exercise intensities were comparable to CON, and crucially, remain well below the rupture threshold for aneurysmal tissue (800–1,200 kPa; 61–83 and 64–83% below for ascending and descending aorta, respectively). Moreover, the values we measured for aortic wall thickness differ less than 5% from published normative values using similar MRI methodology, 7 12 and the values for aneurysmal diameters aligned with health records for our patient population; thus, we are confident in our measures of AWS. This provides preliminary, but compelling evidence as to the safety of acute light-to-moderate intensity aerobic exercise in individuals with ATAA.

Current exercise and physical activity recommendations for individuals with ATAA encourage light-to-moderate aerobic exercise (i.e., 3–5 METS 1 3 ), on the basis that this may be sufficient to improve cardiovascular health and function without exposing the aorta to excessive AWS that could be generated by higher intensities of exercise. 1 However, this recommendation is largely based on expert opinion rather than direct physiological evidence. Therefore, our study provides important evidence to inform future exercise prescription guidelines. Firstly, we have demonstrated the utility of an approach that combines key determinants of AWS (i.e., systolic BP, aortic diameter, and wall thickness) to provide a more wholistic representation of the hemodynamic stress aerobic exercise places on the aorta. Current exercise guidelines suggest exercise BP assessment could help inform exercise prescription in individuals with ATAA (and other thoracic aortic diseases), 3 yet no clear definitions of safe or unsafe values have been provided, nor does this recommendation account for the vastly different AWS values that could be obtained in individuals with the same systolic BP but differing aortic diameters or wall thicknesses. In contrast, studies using uniaxial or biaxial strength testing of healthy and aneurysmal aortic tissue samples have shown a circumferential AWS of 800 to 1,200 kPa is required to rupture an aneurysmal aorta (compared with ∼2,000 kPa for healthy aortic tissue). 7 Therefore, our finding that the ascending circumferential AWS values obtained in our ATAA group at moderate intensity exercise were between 198 and 312 kPa provides preliminary support to the belief that acute “light-to-moderate” intensity exercise is safe for patients with ATAA. This also provides a potential framework for more individualized exercise prescription than determining an arbitrary BP cutoff for all individuals. The patients with ATAA reported here had excellent BP management, which may have attenuated the AWS response during exercise; however, the current exercise recommendations are specific to persons with well-controlled BP. Future work in patients with ATAA with poorly managed hypertension is a necessary next step in this line of inquiry.

**Fig. 4:**
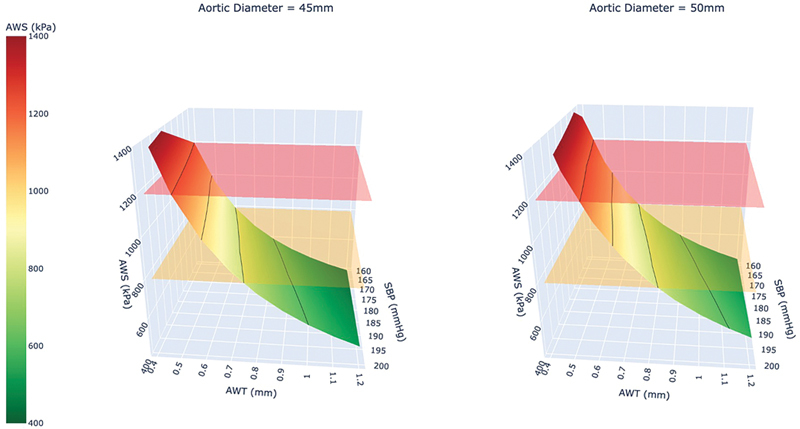
A three-dimensional scanner plot highlighting that a dilated or aneurysmal aorta with a thin wall when combined with an acute and marked increase in systolic blood pressure may result in a ‘perfect storm’ that increases the risk of aortic dissection when AWS surpasses the maximum tensile strength of a fragile aorta. AWT, aorta wall thickness; SBP, systolic blood pressure.

There are still concerns that higher intensities of exercise may expose the dilated thoracic aorta to unsafe levels of AWS. 1 We did not measure circumferential AWS responses at higher intensities of exercise, so cannot determine the values that would be generated, nor the acute safety of higher intensities of exercise in this patient group. However, if we impute the systolic BPs measured at maximal upright exercise (during cardiopulmonary exercise testing) alongside the aortic diameters and wall thicknesses measured during the exercise CMR assessment, the circumferential AWS in the ATAA group would be approximately 277 kPa [211–457], with the highest values measured in our patient who collectively had the highest systolic BP (199 mm Hg), largest aortic diameter (51.7 mm), and thinnest wall (1.5 mm). While this is still below the theoretical rupture threshold of approximately 800 to 1,200 kPa, 7 it illustrates the importance of understanding the concomitant changes in all components of AWS, as this individual's peak AWS was approximately 1.5- to 2-fold higher than the other two patients with ATAA, yet their peak systolic BP was only 54 (38%) and 13 mm Hg (7%) higher, respectively. Indeed, if we consider the conditions necessary to reach critical circumferential AWS values that could cause the aortic wall to rupture (i.e., 800–1,200 kPa), this situation might arise in an aneurysmal aorta (50 mm) at a BP between 170 and 180 mm Hg when the aorta wall thickness is between 0.5 and 0.7 mm. For a dilated aorta (45 mm), this could occur at a BP range of 190 to 200 mm Hg with a wall thickness of 0.5 to 0.7 mm ( Fig. 4 ). This integrative approach might explain why aortic dissections can occur in aortas ≤ 5.0 cm, especially under a “perfect storm” scenario where a dilated and thinned aortic wall faces a significant and marked rise in circumferential AWS that surpasses the maximum tensile strength of the vulnerable aorta ( Fig. 4 ).

Despite similar absolute resting and exercise AWS values, the patients with ATAA in our case series exhibited potentially important differences in cardiovascular function when cardiac and aortic responses were integrated together using the AWS-Qc slope ( Fig. 3B ). Rapid increases in AWS are considered a potential stimulus for aortic wall rupture and/or dissection. 1 An increased AWS-Qc slope may therefore be problematic in individuals with ATAA, as even mild-to-moderate increases in Qc (as one may expect with basic activities of daily living such as running for the bus) could result in rapid relative increases in AWS. The higher AWS/Qc slope values (i.e., reduced aortic mechanical efficiency) observed in patients with ATAA is likely driven by lesser Qc augmentation and/or elevated systemic vascular resistance—both of which could be explained by the lower VO _2_ peak in the ATAA group. This suggests that beyond absolute AWS (and other metrics of aortic function), an additional consideration in ATAA may be global cardiovascular deconditioning, in which the lesser ability to augment Qc and/or decrease vascular resistance during exercise places exaggerated stress on the aortic wall. Notably, increased VO _2_ peak and/or regular exercise training is associated with improvements in both Qc augmentation and, in particular, decreased systemic vascular resistance during submaximal exercise 13 —raising the potential of regular exercise training to improve cardiac and vascular health and lower chronic stress on the aorta.

**Fig. 5:**
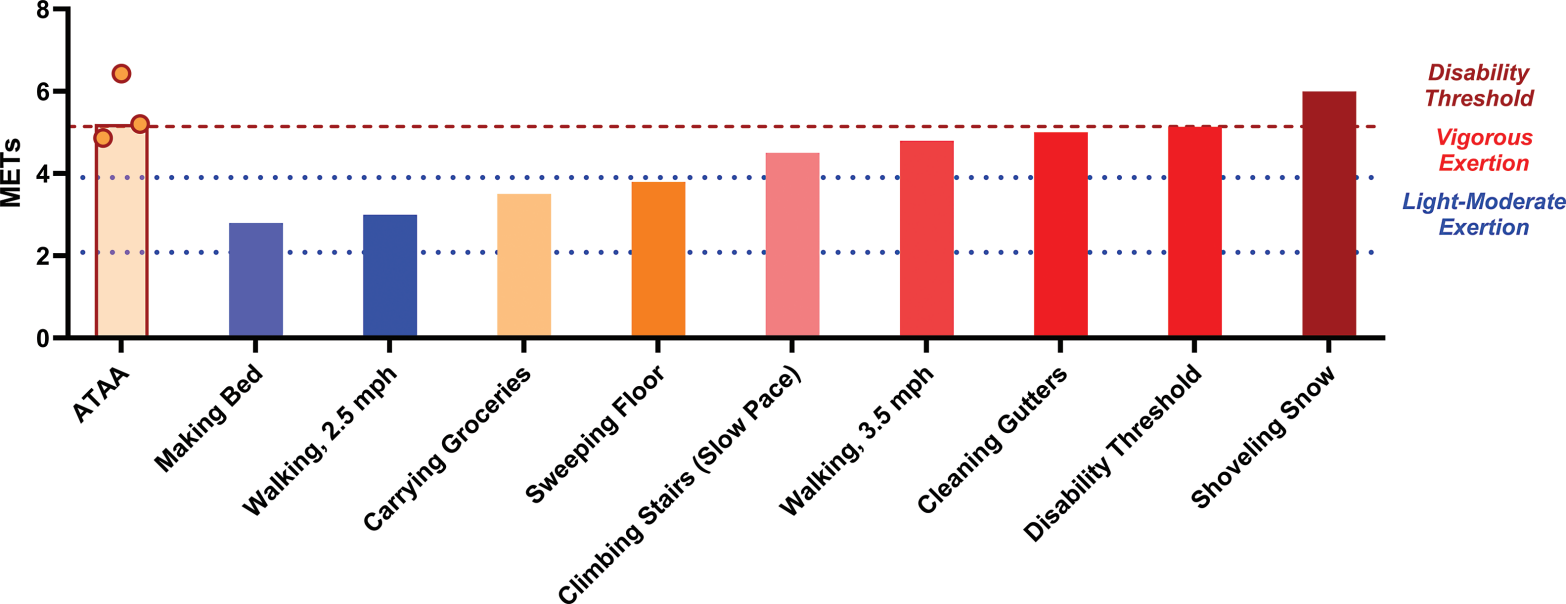
Metabolic equivalents (METs) of various activities of daily living relative to maximal MET values for individuals with an ascending thoracic aortic aneurysm. The red dashed line corresponds to the median VO _2_ peak value measured in the thoracic aortic aneurysm case series.

The relatively low VO _2_ peak we measured in the patients with ATAA (17.0–22.5 mL/kg/min) raises the potential consequences of sedentary behavior that could be induced by exercise restriction, as decreased VO _2_ peak is linked to increased all-cause and cardiovascular mortality. 2 The low VO _2_ peak values also introduce additional considerations for exercise prescription in this cohort. Indeed, in agreement with larger observational studies assessing VO _2_ peak in individuals with ATAA and/or thoracic aortic dissection, 14 15 we found that our three individuals with ATAA VO _2_ peak was typically lower than CON, and for two of them, was at or below the VO _2_ required for full and independent living. 11 This highlights that an arbitrary exercise intensity recommendation of 3 to 5 METs may be sufficient for some, insufficient for others (e.g., those with a peak capacity > 9 METs—such as the endurance athlete with a dilated aorta 16 ) or could be excessive for many with reduced VO _2_ peak 14 15 —where 5 METs (equivalent to a VO _2_ peak of 17.5 mL/kg/min) is equivalent to, if not above, their maximum exercise capacity ( Fig. 5 ). Taken together, this highlights the importance of exercise prescription that incorporates each individual's VO _2_ peak and aortic hemodynamics.

Despite the small sample size, a key strength of our investigation is that, to our knowledge, it is the first measurement of thoracic AWS during exercise. Our findings provide the first physiological evidence supporting the safety of the recommendations for people living with aortic disease, specifically those with ATAA who may be at greatest risk. However, it is important to note that our observations were limited to the guideline-recommended “light-to-moderate” intensity/3 to 5 MET range. 1 Taken together, these findings represent an important first step toward developing evidence-based, individualized exercise prescriptions for individuals with ATAA, but also emphasize the need to understand how AWS responses differ at moderate intensity exercise using more individualized thresholds or definitions. Indeed, while our results support the safety of current exercise guidelines, they also highlight the need for a more nuanced approach that considers individual cardiorespiratory fitness levels, BP responses, and aortic geometry when prescribing aerobic exercise intensity.

### Future Directions

Future studies are warranted, firstly to examine AWS responses on a larger scale to confirm our preliminary and largely exploratory findings. However, the low circumferential AWS values we measured also raises the need to assess thoracic AWS responses during higher absolute intensities of exercise (i.e., >5 METs), which may also be encountered in daily life, and potentially more appropriate for selected patients with higher VO _2_ peak values—where moderate intensity exercise (relative to their higher VO _2_ peak) will reflect higher MET values. This will be important to understand whether exercise recommendations (and restrictions) should be based on the basis of a relative or absolute intensity of exercise to ensure more individualized exercise prescription. Our study also focused on responses to acute exercise; however, there is also the possibility that repeated exercise exposure (e.g., with structured exercise training) could result in changes to aortic structure and function—either favorable or unfavorable. Therefore, there is a need for studies to understand how changes in AWS exercise responses evolve over time, if thoracic AWS values generated by light-to-moderate intensity exercise are sufficient to cause structural or functional remodeling, and whether any metrics (either AWS, or the AWS-Qc slope) can predict future changes in other metrics of aortic structure and function. Ultimately, we speculate that this approach may have useful clinical applications by (1) helping to develop individualized protocols for exercise prescription and restriction based on AWS values and (2) providing additional metrics of aortic function that may improve risk stratification and/or prediction of adverse aortic events—particularly those related to exercise exposure.

## Conclusion

In clinically stable patients with ATAA who have well-controlled resting BP, this first case series report on thoracic AWS (ascending and descending) during guideline-recommended aerobic exercise demonstrates that the increase in AWS is similar between patients with ATAA and CON and remains well below values that have been implicated in aortic wall rupture. The measurement of exercise AWS, and AWS/Qc slope may, therefore, be important metrics for prescribing and monitoring exercise and aortic growth in this population. These findings provide initial physiological evidence supporting current exercise guidelines while highlighting the potential for more personalized exercise prescriptions based on individual AWS responses. Future research examining AWS during higher absolute intensities of exercise, and longitudinal changes in aortic remodeling associated with regular exercise training will further refine our approach to exercise prescription for those with ATAA.
